# A qualitative study of barriers and facilitators to the implementation of a pilot school-based, toothbrushing programme

**DOI:** 10.1186/s12903-022-02494-7

**Published:** 2022-10-24

**Authors:** Ming-Ching Wang, Zoe Marshman, Wei-Han Chen, Wen-Yu Shih

**Affiliations:** 1 Division of Dentistry, Taipei Municipal Hospital, WanFang Branch, Wan Fang Hospital, Taipei Medical University, No. 111, Section 3, Xing-Long Road, Taipei, 116 Taiwan; 2 Department of Dentistry, National Yang Ming Chiao Tung University, No.155, Sec.2, Linong Street, Beitou District, Taipei, 112 Taiwan; 3 Department of Stomatology, Taipei Veterans General Hospital, No.201, sec. 2, Shipai Rd., Beitou District, 112 Taipei, Taiwan; 4 School of Clinical Dentistry, The University of Sheffield, 19 Claremont Crescent, Sheffield, S10 2TA UK

**Keywords:** School-based toothbrushing programme, Classmate-supervised toothbrushing, Habit, Fluoride toothpaste, Theoretical Domains Framework (TDF), Qualitative research

## Abstract

**Background:**

While supervised toothbrushing programmes have been established in many countries of the world, little is known about different perspectives on their implementation. The aim of the study was to explore stakeholders' barriers and facilitators to implementation of a school-based toothbrushing programme in Taiwan.

**Methods:**

Focus groups and interviews were used to explore the views of elementary school students, teachers, staff, and nurses in a piloted school-based toothbrushing programme. The topic guides were developed according to the Theoretical Domains Framework (TDF) to cover the behavioural factors systematically and comprehensively. Data were analysed with content analysis.

**Results:**

Overall, 36 students, 29 teachers/staff, and five school nurses (N = 65) were included. The overarching theme was the importance of habit formation for both staff and children to ensure that toothbrushing as part of the programme was embedded into the school schedule and routine. While children did not necessarily appear to retain the dental knowledge which was taught in the programme, the provision of fluoride toothpaste and toothbrush for their use in schools allowing teachers and staff to choose the timing of the brushing and engaging classmates to supervise each other were found to be key factors.

**Conclusions:**

Implementing a school-based toothbrushing programme with the support of staff and active engagement of children can help children to develop a toothbrushing habit. Classmate-supervised toothbrushing may reduce the burden on teachers and staff to implement the programme.

**Supplementary Information:**

The online version contains supplementary material available at 10.1186/s12903-022-02494-7.

## Background

Dental caries, a highly preventable disease, is the most prevalent chronic disease in children [1, 2]. The high proportion of untreated caries in children is particularly problematic (in terms of pain, infection, difficulty eating, perhaps sepsis, and educational attendance), which makes prevention even more critical [3]. Toothbrushing with fluoride toothpaste has been shown to be highly effective at preventing childhood caries [4, 5]. Ideally, home-based parent-supervised twice-daily brushing with fluoride toothpaste is recommended [6]. However, for some families, optimal home-based oral health behaviours may be difficult to achieve [7, 8], which has the potential to further widen inequalities in oral health. Children from the lowest socio-economic conditions bear the greatest burden from caries [9, 10]. These impacts are worth emphasising from both health and education perspectives.

Therefore, school-based toothbrushing programmes are recommended as a community-based oral health promotion strategy [11]. Daily supervised toothbrushing with toothpaste containing more than 1000 ppm fluoride in nurseries and schools was found to effectively reduce caries and reduce oral health inequalities [9, 12, 13]. This intervention also has the potential to be inclusive of both children who do and do not routinely access dental services. Indeed, when the costs of implementing a toothbrushing programme as part of the Childsmile programme were compared to the cost of dental treatment, the expected long-term savings to the health service have been estimated to be more than two and a half times the cost of the toothbrushing programme [13].

However, little research has been conducted into the practical implementation of these school-based toothbrushing programmes or their impact on oral health knowledge, skills, or longer-term behaviours, including daily brushing with fluoride toothpaste [14]. More research into the behavioural aspects of this intervention has been recommended [15, 16], including the use of behaviour change theory [17].

Human behaviours are complicated, and they have been addressed by many behaviour theories [18, 19]. Various theories cover different behavioural factors, and hence, these theories have different strengths and weaknesses. The Theoretical Domains Framework (TDF) is derived from 33 psychological theories and includes 14 theoretical determinants of behaviour, namely: Knowledge', 'Skills', 'Social/Professional Role and Identity', 'Beliefs about Capabilities', 'Optimism', 'Beliefs about Consequences', 'Reinforcement', 'Intentions', 'Goals', 'Memory, Attention and Decision Processes', 'Environmental Context and Resources', 'Social Influences', 'Emotions', and 'Behavioural Regulation'. The TDF can be used to analyze problems and design interventions in a diverse and systematic way [20]. The TDF has been applied to a study of parent-supervision of young children's toothbrushing and was found to be an appropriate and comprehensive framework with all domains relevant [21]. The TDF has also been used to identify barriers and facilitators to sexual health service use among university students and the TDF domains (memory, attention and decision-making processes; social influences; environmental context and resources; beliefs about consequences, etc.) could be the mechanisms of university students' access of sexual health services. It was suggested that future interventions to change oral health behaviours should be designed and evaluated based on behavior change theory. Then the intervention could be expected to be more effective, and the analysis might be more comprehensive [22].

In Taiwan, the prevalence of caries is high, with 65.4% of 5-year-old-children having caries experience in 2017 [23, 24]. The mean DMFT of 12-year-old children in 2020 was 2.01 [25]. Considering the high prevalence (and severity of caries) in Taiwan, a school-based toothbrushing programme was introduced to supplement home- and clinic-based interventions. The programme was piloted in six elementary school, and before a decision was taken to extend the programme to other areas, an evaluation of the implementation was required [26, 27]. Therefore, the aim of the study was to explore the facilitators and barriers of implementation of a school-based toothbrushing programme with children, teachers, staff, and school nurses. The study was guided by the Theoretical Domains Framework, and the findings would be used to inform the future implementation of a more extensive programme.

## Methods

### Overview

This study uses qualitative methods guided by behaviour change theory to explore the barriers and facilitators of implementation of a pilot school-based toothbrushing programme in Taiwan.

### The pilot toothbrushing programme

There were six schools involved in the piloted school-based toothbrushing programme for 12 months. The children were aged six to twelve years old. These children were asked to brush their own teeth twice daily for five school days a week in the programme [28, 29]. The toothbrushing programme was developed as a complex intervention with multiple components and was implemented to fit the different needs for each school [30].

A paediatric dentist (MCW) engaged with school nurses, teachers, and staff to establish the pilot programme. There was no training session provided for staff although the benefits of caries prevention were explained. School nurses and teachers provided teaching about oral health and toothbrushing according to children's textbooks. Additionally, dental students attended the schools to provide the children with one session of instruction on how to brush their teeth effectively. Teachers were not asked to supervise the children while they brushed, but most teachers supervised of their own will at the beginning of the programme. There was no parental involvement, but all parents provided consent for their children to take in the programme.

The piloted programme aimed to change children's oral health behaviours by improving their capability, opportunity, and motivation according to the COM-B model [31]. For example, toothpaste containing 1000 ppm fluoride was given to the school since most children's toothpaste in Taiwan contains less than 500 ppm of fluoride, which is not as effective in caries prevention (opportunity) [5]. Dental students provided a one-hour teaching session for children on how to brush their teeth systematically, how to use the modified Bass brushing technique and how to choose 1000 ppm fluoride toothpaste for future home use (capability), but no parents were involved. Dental students also tried to motivate children by explaining the health and social consequences of dental caries. The teaching activities included didactic teaching, role-playing, activities, and games according to the age of the children (motivation). A scheme was introduced whereby children could collect points by brushing twice daily with a reward for a month of participation in the programme (motivation).

There was no standard protocol or manual used; each school decided how they preferred to implement the pilot programme, such as the timing of toothbrushing or whether there would be adults to supervise children brushing. Schools were free to choose aspects of the intervention, such as whether to play a brushing song or use a bell as a cue to remind children it was time to undertake to toothbrush.

### Study procedure

The study procedure was guided by the TDF and involved the following six steps [32]:

1. Selection of the target behaviour:
  In order to decrease dental caries in children, implementing the school-based toothbrushing programme was selected as the target behaviour [28, 29].
2. Selection of the study design
  Qualitative interviews were chosen to explore potential barriers and facilitators of the implementation of the toothbrushing programme from the different stakeholder's perspectives [32]. Focus groups and one-to-one interviews were used depending on the preferences of the participants and to provide both a breadth of data and a depth of data, respectively [33]. The locations were chosen by schools for their convenience (classrooms, meeting rooms, or libraries).
3. Development of the study material
  A topic guide for the semi-structured qualitative interviews was developed based on the literature about the school-based toothbrushing programme [21, 34–36] and moderated by an experienced paediatric dentist with qualitative research training (MCW and WHC) and they took notes during data collection. Open questions were designed specifically with each theoretical domain to cover the programme comprehensively [22, 37, 38]. The order in the TDF was not strictly followed to keep the flexibility for the response of participants. The topic guide was piloted with other children and teachers to ensure the questions were clear and understandable for elementary school students. All participants' unclear words or ideas were clarified in the interview directly to increase the interpretative validity [39, 40]. All sessions were audio-recorded for transcription and analysis. Ethical approval was obtained from Taipei Veterans General Hospital Research Ethics Committee (Approval No.: 2017-06-012B).
4. Development of the sampling strategy
  A purposive sample of elementary students, teachers, staff, and school nurses was chosen. Children, teachers, staff, and school nurses are the primary stakeholders inside the school, and they play important roles at individual, interpersonal, and institutional levels. Different groups of participants were expected to bring maximum variations of the responses [32]. Participants' descriptions were collected as much as possible to increase the richness of the data. Data saturation was deemed to have been reached when three consecutive participants did not provide additional ideas [41].
5. Data collection
  All the participating children, teachers, and staff were invited by school nurses to share their experiences, feelings, and how they would improve this toothbrushing programme. All discussions were arranged at least three months after the initiation of the programme to ensure the participants had enough ideas about the programme. The time was determined by the school or participants' schedules. Their discussions were guided by the TDF-based topic guide. The flexibility was kept during the discussion to reflect participants' daily situations [22]. Twenty minutes has been deemed the minimum to allow the participants to express their opinions. Participants' unclear words or ideas were confirmed in the interview directly to increase the interpretative validity [39, 40]. All sessions were audio-recorded for transcription and analysis.
6. Data analysis

The data were analyzed using content analysis [42]. Coding was done by extracting meaningful units related to the target brushing behavior from the data. The codes were preliminarily grouped into barriers or facilitators. Then, the codes were categorised into 14 themes based on the domains of the TDF (Table 1) [43, 44]. Their operational definitions were derived from the American Psychological Associations' Dictionary of Psychology [43, 44]. For example, the coding rules of 'Environmental context and resources' domain could be toothpaste, toothbrushes, or schedules which encourage children' toothbrushing behaviour. The codes might be allocated to one or more domains according to their meanings [32].

**Table 1:** Themes (domains from the Theoretical Domains Framework [43]), in the content analysis of the barriers and facilitators

TDF domain (Theme)	Definition	Barrier from interview	Facilitator from interview
Knowledge	An awareness of the existence of something	“*Someone came to teach us about brushing. But I don't remember exactly the name of the method of brushing… Something that starts with a B…. We watched a video about it*” – Student	*“The story book told us that brushing daily can prevent teeth from decaying.”* - Student
Skills	An ability or proficiency acquired through practice	–	*“I want someone to confirm whether I brush properly or not.”* - Student
Social/professional role and identity	A coherent set of behaviours and displayed personal qualities of an individual in a social or work setting	*“School is a place for education. There is something needing to be covered in each session. In addition to oral hygiene issue, there are also drug issue, physical education. … Students need to apply what they have learned. They should brush themselves at home, instead of doing everything at school” *- *Teacher*	*“What said by teachers to children is very influential.”* - Deputy head teacher
Beliefs about capabilities	Acceptance of the truth, reality, or validity about an ability, talent, or facility that a person can put to constructive use	*“It may be too difficult for children under 7 to do it (flossing) by themselves, but children above 8 could give it a try.”* - Teacher	*“The problem with brushing skill can be solved by teaching in lectures. The difficulties in children’s brushing skill can be managed by teaching sessions. Children are all trainable.”* - Teacher
Optimism	The confidence that things will happen for the best or that desired goals will be attained	*“I will continue to brush when I become a junior high school student, but I may not be able to brush in the morning.”* - Student *“Brushing twice daily does not place much burden on schools.”* - Deputy head teacher	*“Children do not think about whether they brushing properly or not. Children usually think they know how to brush, and they are brushing pretty well.”* - Teacher
Beliefs about consequences	Acceptance of the truth, reality, or validity about outcomes of a behaviour in a given situation	*“If I do not brush, I am afraid that I will have cavities. However, I have no idea what would happen if I have cavities.”* -Student	*“I am afraid that my teeth may get yellowish or decayed. This makes me want to brush after eating.”* - Student
Reinforcement	Increasing the probability of a response by arranging a dependent relationship, or contingency between the response and a given stimulus	*“Children can get gifts as they brush. However, I am afraid that children will only brush when they can get gifts later in life.”* - Teacher	*“The red thing (disclosing agent) looks funny, we can have a competition with gift about it.”* - Student
Intentions	A conscious decision to perform a behaviour or a resolve to act in a certain way	“Sometimes, *I am so tired during the lunch break so I do not brush.*” – Student “The reason of skipping brushing is slacking off.” – Teacher	–
Goals	Mental representations of outcomes or end states that an individual wants to achieve	*“Brushing twice a day to gain points for gifts can be implemented well by children. However, the reason of brushing became the gifts.”* – Teacher	*“I want to brush even more, if I can collect the points for gifts.”* - Student
Memory, attention and decision processes	The ability to retain information, focus selectively on aspects of the environment and choose between two or more alternatives	“*I do not remember that there were people (dental students) who came to teach us brushing.*” - Student	*“When I buy toothpaste, I occasionally want to buy the one I got from school.”* - Student
Environment context and resources	Any circumstance of a person's situation or environment that discourages or encourages the development of skills and abilities, independence, social competence, and adaptive behaviour	*“There are only 5 min in the other two breaks in the morning. Students do not have enough time to brush.” *- Teacher	*“Students would not spend money buying oral hygiene kit, but they would use it if the kits were provided free.” *- Teacher
Social influences	Those interpersonal processes that can cause individuals to change their thoughts, feelings, or behaviours	*“My parents remind me to brush all the time, but they do not brush with me.”*—Student	*“One student would get another student to brush. They would remind each other to brush.”*—Teacher
Emotion	A complex reaction pattern, involving experiential, behavioural, and physiological elements, by which the individual attempts to deal with a personally significant matter or event	*“It should be not too embarrassing to brush in front of classmates.”* – Teacher	*“It is fun to see other people brushing.”* – Student
Behavioural regulation	Anything aimed at managing or changing objectively observed or measured actions	*“We won’t remind each other to brush during winter and summer vacation.”* *-* Student	*“The brushing song (which reminds children to brush) would be played at the brushing time *- Teacher

The coding was carried out by WHC. Codes were categorised into different domains by MCW and WHC with discussion with ZM to establish consensus [45] and reduce the interpreter bias [46]. This whole paper was written with COREQ statement (Additional file 1).

## Results

### Overview of the participants

Participants were recruited from the six schools involved in the pilot programme who had implemented the toothbrushing programme for at least three months. We conducted 11 focus groups and 11 interviews, including 36 children, 29 teachers and staff (including 3 deputy head teachers and 1 principal), and 5 school nurses (N = 65).

The barriers and facilitators of implementing the toothbrushing programme were reported according to the 14 domains in the TDF (Table 1).

### Memory, skill, knowledge and beliefs about the consequences in children

The results from the domains of memory, skill, knowledge, and beliefs will be described together as these related closely to each other.

The barriers to memory domains were: children could not remember what was taught by the dental students, and some children had completely forgotten the dental students had even visited, despite the dental students employing engaging activities (cartoons, role-playing, and games). It may be that one single session was insufficient. Nevertheless, children did have some dental knowledge and brushing skill which may have resulted from their own school teachers.

*"It is good to have college students to give dental education, but the children did still forget after a while."* – Deputy head teacher

### Professional role and priorities about the toothbrushing programme

Some teachers and staff did not think it was their job to prevent dental caries, and that their professional role was educating children. Consequently, these teachers and staff may have prioritized other activities and be less likely to integrate the toothbrushing programme into the school schedule. The children may also have other priorities that make them miss their brushing schedule such as sport or art activities.

*"There is a set time to brush at lunchtime but there is no such time in the morning. Children need to tidy the classroom, read and eat breakfast."* – Teacher

*"Some children just do not remember to brush. They may have other activities or plans, and this makes them forget."* – Teacher

### Behavioural regulation, environmental context and reinforcement of the toothbrushing routine

The programme was designed to improve children's brushing behaviours, so it was carried out regularly at school. In some schools, the brushing song or bell was played at the same time every day to encourage children to form the brushing routine. The data suggested that over time teachers felt they no longer needed to supervise children and that the brushing song or bell could alone be a suitable cue for children to brush their teeth. Indeed, they suggested that children begin to supervise each other as the brushing routine becomes a habit. Going one step further, it was even suggested that children might even regulate their teacher's behaviour to let them brush.

*"Young children, they are not yet able to understand (the importance) and, hence, motivate themselves (to brush their teeth). They go to brush their teeth as they hear the brushing song."* - Teacher

*"Children above eight (are old enough) and already have the habit of brushing. If they do not brush, their classmate would remind them, "how come you do not brush? That is so disgusting!"."* - Teacher

*"Children have got used to this activity in their lives. They may even remind the teachers to let them brush when the time comes" -* Teacher

### Social influence of classmate-supervised toothbrushing

Children's brushing behaviour was influenced by their classmates. Data showed that classmates reminded each other to brush and indeed, both teachers and children suggested children did not need to be supervised by teachers. At this age, six to twelve years old, teachers felt the children's brushing habits could be easily cultivated.

*"They see the whole class are brushing so they start brushing. The most important thing is to cultivate good habits as soon as possible. It is like washing their hands after using the toilet."* - Teacher

### Environmental resources to encourage toothbrushing behaviours

The data suggested that many parents did not buy suitable toothpaste or toothbrushes for their children to brush at school. Some parents forgot to buy the brushing kit when it was time for it to be replaced. It was suggested that it is easier and potentially more clinically effective to run the programme where the necessary resources of toothpaste and toothbrushes are provided to ensure they are appropriate and always available to all children.

*"Children need toothbrush and toothpaste, and these need to be provided by the schools rather than their parents. Parents are sometimes not that cooperative." –* Teacher

**Fig. 1:**
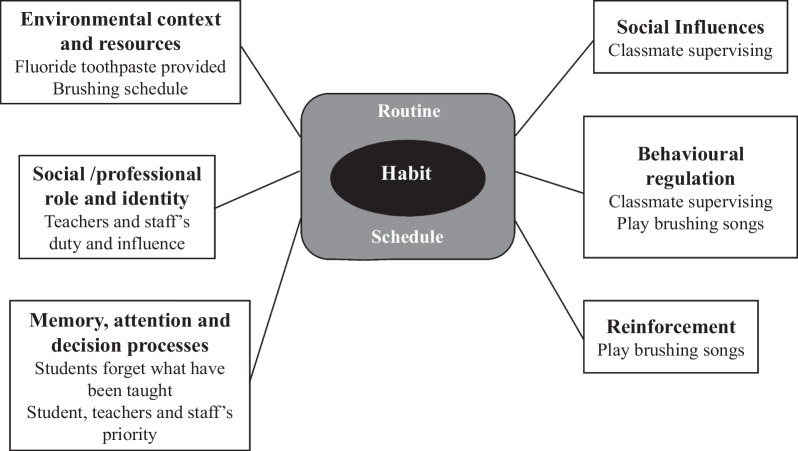
Habit is the overarching theme for successful implementation of the school-based toothbrushing programme. The six main themes from the TDF: social influences; environmental context and resources; behavioural regulation; reinforcement; social professional role and identity; memory, attention and decision processes

After further analysis, habit was viewed as being an overarching theme to the successful implementation of this toothbrushing programme. School routine and schedule in the environment reinforced habit formation which relates to classmate-supervised toothbrushing with social influence and behavioural regulation (Fig. 1).

## Discussion

The aim of this study was to explore the barriers and facilitators of implementing a pilot toothbrushing programme in Taiwan. We gathered information from the primary stakeholders in the toothbrushing programme- children, teachers, staff, and school nurses. The results of the implementation the programme were similar across different schools though their protocols may have been different. The identification of the barriers and facilitators was guided by the TDF with the main themes reflecting the domains of: social influences; reinforcement; behavioural regulation; environmental context and resources; social professional role and identity; memory, attention and decision processes. The overarching theme for the implementation of this programme was habit, suggesting school routine and schedule could be effective facilitators.

The study highlighted an interesting finding about the role of teachers and supervison in toothbrushing programmes. Some teachers felt their professional role was to educate children rather than prevent caries in children. Teachers in a previous study also suggested that the responsibility for supervising children's brushing should fall to parents rather than teachers, and some of our teachers also thought so [47]. In our study, one suggestion from teachers was that once a habit was established, they did not need to supervise children and that classmates could supervise each other to brush. This raises important questions about the responsibilities of school staff and whether children can or should be left to regulate their own behaviours, including toothbrushing. It may be that social influences among children can be effective facilitators in implementing this toothbrushing programme, and giving children an active role may increase engagement. Further exploration of the supervision of toothbrushing programmes is needed as what is appropriate may vary from country to country and depending on the age and abilities of the children involved. Students might even supervise their teacher to let them brush when the brushing time was coming. Therefore, students were perceived as no longer taking a passive role in the toothbrushing programme.

In the environment domain, the provision of brushing kits as part of the programme is important. Most children's toothpastes in Taiwan market do not contain more than 1000 ppm fluoride. In this study, many teachers and nurses mentioned that parents did not provide suitable toothpaste for their children hence limiting their opportunities to undertake toothbrushing at school. This may imply that some children also do not have access to 1000 ppm fluoride toothpaste at home, either. Hence, it may be more practical to provide suitable toothpaste at school provided by the programme directly. In a UK study, parents with high-caries-risk children mentioned that free toothpaste and toothbrush benefitted their families, and the cost of toothbrushes and toothpaste was a potential barrier to regular toothbrushing. Providing brushing kits is recommended to help schools establish effective children's toothbrushing [48].

Motivation for toothbrushing for this young age group can be difficult, but it is potentially a good time developmentally to cultivate a toothbrushing habit [49]. In this pilot programme, we tried to teach the children the potential benefits of brushing and the consequences of not brushing. However, providing knowledge alone is not sufficient to develop motivation. However, we did find the children easily got used to the brushing habit when brushing time was established in the school schedule (environmental context and resources domain). Children were found to brush in response to the cue of hearing the brushing song played (reinforcement domain) which appeared to become a habit, rather than for reasons of health improvement (knowledge or beliefs about consequences domains) (Fig. 1).

Studies have shown that dentists visiting school settings to teach children or pure dental education may not be effective [28, 50, 51]. In this study, children do not remember what dental knowledge was taught by dental students. Children did not even remember that there were dentists or dental students who came to teach them. However, children did seem to recall what their teachers taught them about brushing (knowledge or memory, attention and decision processes domains). It may be that teachers understand better how to design and deliver sessions to children and have the opportunity to reinforce the knowledge and skills regularly. The literature would suggest that while dental expertise is needed to inform the content of teaching about oral health, dental professionals do not need to teach children directly [11, 51] or indeed deliver oral health messages to other patient groups. Dentists' role in these programmes is to provide teachers with accurate knowledge to prevent teachers from gathering wrong information or providing outdated knowledge (social/professional role and identity domain) [11, 52]. Another previous paper also showed that dentists need to provide correct knowledge to primary health care workers who are pregnant women and mommies with babies to meet and get information regularly [53].

The routine brushing time was important to habit formation, and this also made it easier for these children to develop the habit of brushing regularly and consistently. Previous papers have also mentioned that brushing should take place daily, fitting into the schedule when it is most convenient for the classroom schedule [54]. In the pilot programme, the teachers were able to decide which were the two feasible brushing timings according to their own school's schedule. The flexibility of schedule decreased the inconvenience of teachers and staff and perhaps increased their motivation to participate in the programme. However, as mentioned previously, oral health may not be considered a priority for staff because of their high workload and time constraints [53]. As staff engagement is critical, was is an important factor when implementing any school-based toothbrushing programmes, particularly when doing so on a large scale. Hence, instead of motivating children directly, future intervention designs perhaps should consider how to motivate teachers and staff to help students developing brushing habit. This may improve further implementation or scaleup of school-based toothbrushing programmes.

This study has several limitations. First, it involved convenience sampling. The recruitment of children was carried out by the school nurses rather than the researchers who may have limited the range of views obtained. These children were willing to explain their ideas to researchers, and they did provide abundant information. Nevertheless, the participating children may have shown better compliance and different views than their classmates. We did, however try to confirm what children had said with their classmates during the interviews and focus groups on increasing the interpretative validity [39, 40]. Additionally, we tried to recruit children from different schools and ages to improve the range of views. We also tried to increase the validity through data triangulation, so teachers, staff, and nurses were all invited to be participants in interviews which were held individually and in groups [33]. While the increases the complexity of the data collection and analysis, it will also provide a more comprehensive overview of barriers and facilitators across the whole school setting. There may be some minor inconsistent codings in the data brought from one-on-one interviews or focus groups, but the variations did not affect the main and overarching themes [39, 40].

Another limitation of the study was that the original aim was to explore the implementation of the piloted programme. This programme was a complex intervention tailored to the needs of elementary schools in Taiwan and was relatively recently implemented. While the importance of findings about the overarching theme of habit and of the importance of engagement of staff and children will be generalizable to the implementation of toothbrushing programmes elsewhere, other factors may be important as programmes are introduced in different countries and may be influential for long-term sustainability.

## Conclusion

Overall, this theoretically-informed study revealed significant findings about the implementation of toothbrushing programmes from the perspectives of key stakeholders. There were barriers and facilitators from six domains that formed the main themes: environmental context and resources; reinforcement; behavioural regulation; social influences; social professional role and identity; memory, attention and decision processes. The overarching theme for the successful implementation of a programme was found to be the establishment of habit. Further research is needed to explore the implementation of this important public health intervention for improving children's oral health.

## Supplementary Information

**Additional file 1**. COREQ Checklist.

## Data Availability

The datasets analysed in the current study are available from the corresponding author on reasonable request.

## Abbreviation

TDF: Theoretical Domains Framework
