# Supplementary material for: A qualitative study of barriers and facilitators to the implementation of a pilot school-based, toothbrushing programme
Source: BMC Oral Health. 2022 Oct 24;22:451. doi: 10.1186/s12903-022-02494-7 (PMC9590216; doi:10.1186/s12903-022-02494-7)
Supplement: Supplementary file 1 — Additional file 1. COREQ Checklist. [file 12903_2022_2494_MOESM1_ESM.docx]

**Additional file 1. COREQ Checklist**

| Domain 1: Research team and reflexivity |  |  |  |
| --- | --- | --- | --- |
| Personal Characteristics |  |  |  |
| 1 | Interviewer/facilitator | Which author/s conducted the interview or focus group? | The author conducted all interviews and focus groups. |
| 2 | Credentials | What were the researcher's credentials? E.g. PhD, MD | The author is a PhD student with 15 years clinical experience. Dental public health master degree. |
| 3 | Occupation | What was their occupation at the time of the study? | Clinical paediatric dentist and part-time PhD student. |
| 4 | Gender | Was the researcher male or female? | Female |
| 5 | Experience and training | What experience or training did the researcher have? | 15 years clinical work with high-caries-risk children. The thesis of the author’s master degree is a qualitative study. Training of designing and conducting focus group |
| Relationship with participants |  |  |  |
| 6 | Relationship established | Was a relationship established prior to study commencement? | The researcher (Min-Ching, Wang) visited these schools and did the dental education before the study. However, the researchers only had private connection with the school nurses but not other participants. All student participants were invited by the school nurses. Teachers, staff, and school nurses in the piloted school-based toothbrushing programme were invited and they came if it is convenient for them. |
| 7 | Participant knowledge of the interviewer | What did the participants know about the researcher? e.g. personal goals, reasons for doing the research | All the participants knew the interviewer ran the piloted programme. Participants knew the interviewer wanted to know how they think about the piloted toothbrushing program, and their responses may help to improve the programmed at the same time in addition to this study itself. |
| 8 | Interviewer characteristics | What characteristics were reported about the interviewer/facilitator? e.g. Bias, assumptions, reasons and interests in the research topic | The interviewer is also the one who in charge of the toothbrushing programme, and hence, she may be bias about her own work. Therefore, the second author participated the interviews and did all the analysis with the interviewer to ensure the interviewer did not misunderstand the participants and increase the data triangulation. |
| Domain 2: study design |  |  |  |
| Theoretical framework |  |  |  |
| 9 | Methodological orientation and Theory | What methodological orientation was stated to underpin the study? e.g. grounded theory, discourse analysis, ethnography, phenomenology, content analysis | Content analysis |
| Participant selection |  |  |  |
| 10 | Sampling | How were participants selected? e.g. purposive, convenience, consecutive, snowball | Purposive |
| 11 | Method of approach | How were participants approached? e.g. face-to-face, telephone, mail, email | face-to-face |
| 12 | Sample size | How many participants were in the study? | 65 participants |
| 13 | Non-participation | How many people refused to participate or dropped out? Reasons? | No |
| Setting |  |  |  |
| 14 | Setting of data collection | Where was the data collected? e.g. home, clinic, workplace | Campus. |
| 15 | Presence of non-participants | Was anyone else present besides the participants and researchers? | One public health student (not included in the researchers list) participated as assistant present in the interviews |
| 16 | Description of sample | What are the important characteristics of the sample? e.g. demographic data, date | The student participants were invited by the school nurses rather than the researchers, so they maybe more compliant than other students. However, we deemed them as community champion in the campus. |
| Data collection |  |  |  |
| 17 | Interview guide | Were questions, prompts, guides provided by the authors? Was it pilot tested? | Topic guide was demonstrated in the appendix. It was designed according to the theoretical domains framework. It has been piloted with children of that age group. |
| 18 | Repeat interviews | Were repeat interviews carried out? If yes, how many? | Yes. Two confirmatory interview for rapport. |
| 19 | Audio/visual recording | Did the research use audio or visual recording to collect the data? | Audio recording |
| 20 | Field notes | Were field notes made during and/or after the interview or focus group? | No, but the assistant moderator and the researcher had feedbacks after every session. |
| 21 | Duration | What was the duration of the interviews or focus group? | All the interviews were longer than 20 mins. All the focus groups were longer than 40 mins. |
| 22 | Data saturation | Was data saturation discussed? | Saturation was reached. No new relevant knowledge was obtained after the 14 interview. |
| 23 | Transcripts returned | Were transcripts returned to participants for comment and/or correction? | No |
| Domain 3: analysis and findings |  |  |  |
| Data analysis |  |  |  |
| 24 | Number of data coders | How many data coders coded the data? | One. But the coding and the formation of themes were finished with the two researchers (MCW and WHC). |
| 25 | Description of the coding tree | Did authors provide a description of the coding tree? | Figure show the final themes. |
| 26 | Derivation of themes | Were themes identified in advance or derived from the data? | Derived from the data. |
| 27 | Software | What software, if applicable, was used to manage the data? | Excel, Word |
| 28 | Participant checking | Did participants provide feedback on the findings? | The latter participants from the same school mat provide feedback from the former participants. |
| Reporting |  |  |  |
| 29 | Quotations presented | Were participant quotations presented to illustrate the themes / findings? Was each quotation identified? e.g. participant number | Yes. Demonstrated with their position. |
| 30 | Data and findings consistent | Was there consistency between the data presented and the findings? | Yes |
| 31 | Clarity of major themes | Were major themes clearly presented in the findings? | Yes |
| 32 | Clarity of minor themes | Is there a description of diverse cases or discussion of minor themes? | Yes |
| Table adapted from, Consolidated criteria for reporting qualitative studies (COREQ): 32-item checklist, based on Tong et al. 2007 [1] | | | |

**References**

1. Tong A, Sainsbury P, Craig J: **Consolidated criteria for reporting qualitative research (COREQ): a 32-item checklist for interviews and focus groups**. *Int J Qual Health Care* 2007, **19**(6):349-357.
